# Prevalence of chronic kidney disease and its associated factors in Malaysia; findings from a nationwide population-based cross-sectional study

**DOI:** 10.1186/s12882-020-01966-8

**Published:** 2020-08-14

**Authors:** Thamil Arasu Saminathan, Lai Seong Hooi, Muhammad Fadhli Mohd Yusoff, Loke Meng Ong, Sunita Bavanandan, Wan Shakira Rodzlan Hasani, Esther Zhao Zhi Tan, Irene Wong, Halizah Mat Rifin, Tania Gayle Robert, Hasimah Ismail, Norazizah Ibrahim Wong, Ghazali Ahmad, Rashidah Ambak, Fatimah Othman, Hamizatul Akmal Abd Hamid, Tahir Aris

**Affiliations:** 1 Institute for Public Health, National Institutes of Health, Ministry of Health Malaysia, Shah Alam, Selangor Malaysia; 2 Sultanah Aminah Hospital, Ministry of Health Malaysia, Johor Bahru, Johor Malaysia; 3 Clinical Research Centre Penang Hospital, Ministry of Health Malaysia, George Town, Penang Malaysia; 4 Kuala Lumpur Hospital, Ministry of Health Malaysia, Kuala Lumpur, Malaysia; 5 Selayang Hospital, Ministry of Health Malaysia, Batu Caves, Selangor Malaysia; 6 Tengku Ampuan Rahimah Hospital, Ministry of Health Malaysia, Klang, Selangor Malaysia

**Keywords:** Chronic kidney disease, Prevalence, Associated factors, Adults, Malaysia

## Abstract

**Background:**

The prevalence of chronic kidney disease (CKD) in Malaysia was 9.07% in 2011. We aim to determine the current CKD prevalence in Malaysia and its associated risk factors.

**Methods:**

A population-based study was conducted on a total of 890 respondents who were representative of the adult population in Malaysia, i.e., aged ≥18 years old. Respondents were randomly selected using a stratified cluster method. The estimated glomerular filtration rate (eGFR) was estimated from calibrated serum creatinine using the CKD-EPI equation. CKD was defined as eGFR < 60 ml/min/1.73m^2^ or the presence of persistent albuminuria if eGFR ≥60 ml/min/1.73m^2^.

**Results:**

Our study shows that the prevalence of CKD in Malaysia was 15.48% (95% CI: 12.30, 19.31) in 2018, an increase compared to the year 2011 when the prevalence of CKD was 9.07%. An estimated 3.85% had stage 1 CKD, 4.82% had stage 2 CKD, and 6.48% had stage 3 CKD, while 0.33% had stage 4–5 CKD. Hypertension (aOR 3.72), diabetes mellitus (aOR 3.32), increasing BMI (aOR 1.06), and increasing age (aOR 1.06) were significantly associated with CKD.

**Conclusion:**

Our study has shown that CKD has become one of the leading public health issues in Malaysia. Thus, there is an urgent need to screen for CKD and prevent its progression, associated morbidity, and mortality at the national level.

## Background

Chronic kidney disease (CKD) affects about 1 in 10 adults and accounts for millions of premature deaths worldwide [1–4]. CKD represents a significant public health problem because of the associated high morbidity and mortality, mainly attributable to elevated cardiovascular risk [1, 5, 6]. The National Health and Morbidity Survey (NHMS) in 2011 showed a 9.07% prevalence of CKD in West Malaysia [7, 8]. End-stage renal disease requiring dialysis has shown an increasing trend in Malaysia with an incidence rate of 216 per million population in the year 2016 compared to 96 per million population in 2002 [9]. A total of 37,781 patients were on renal replacement therapy in Malaysia at the end of the year 2016 (at a rate of 1159 per million population) [9], and this consumes a disproportionate amount of our national healthcare budget [10, 11].

It is, therefore, necessary to determine the current prevalence of CKD to assist healthcare planning, resource allocation, and to guide healthcare policy in prevention, early detection, and treatment of CKD. KDIGO’s 2012 Clinical Practice Guideline for the Evaluation and Management of Chronic Kidney Disease supports early detection of CKD in asymptomatic individuals at increased risk [11]. Screening CKD is cost-effective in diabetes mellitus (DM) and hypertensive patients as well as in the general population among the 60-year-old and above [2, 12, 13]. This study aims to determine the current prevalence of CKD among adults in Malaysia and its associated risk factors.

## Methods

This is a cross-sectional study; conducted from September 2017 to June 2018. The target population was residents in non-institutional living quarters (LQ) aged 18 years and above in Malaysia. The Department of Statistics Malaysia provided the latest available sampling frame that is representative of Malaysia, divided into enumeration blocks (EBs) and stratified by the state as well as locality (urban/rural). In 2017, Malaysia had about 83,000 EBs.

**Fig. 1:**
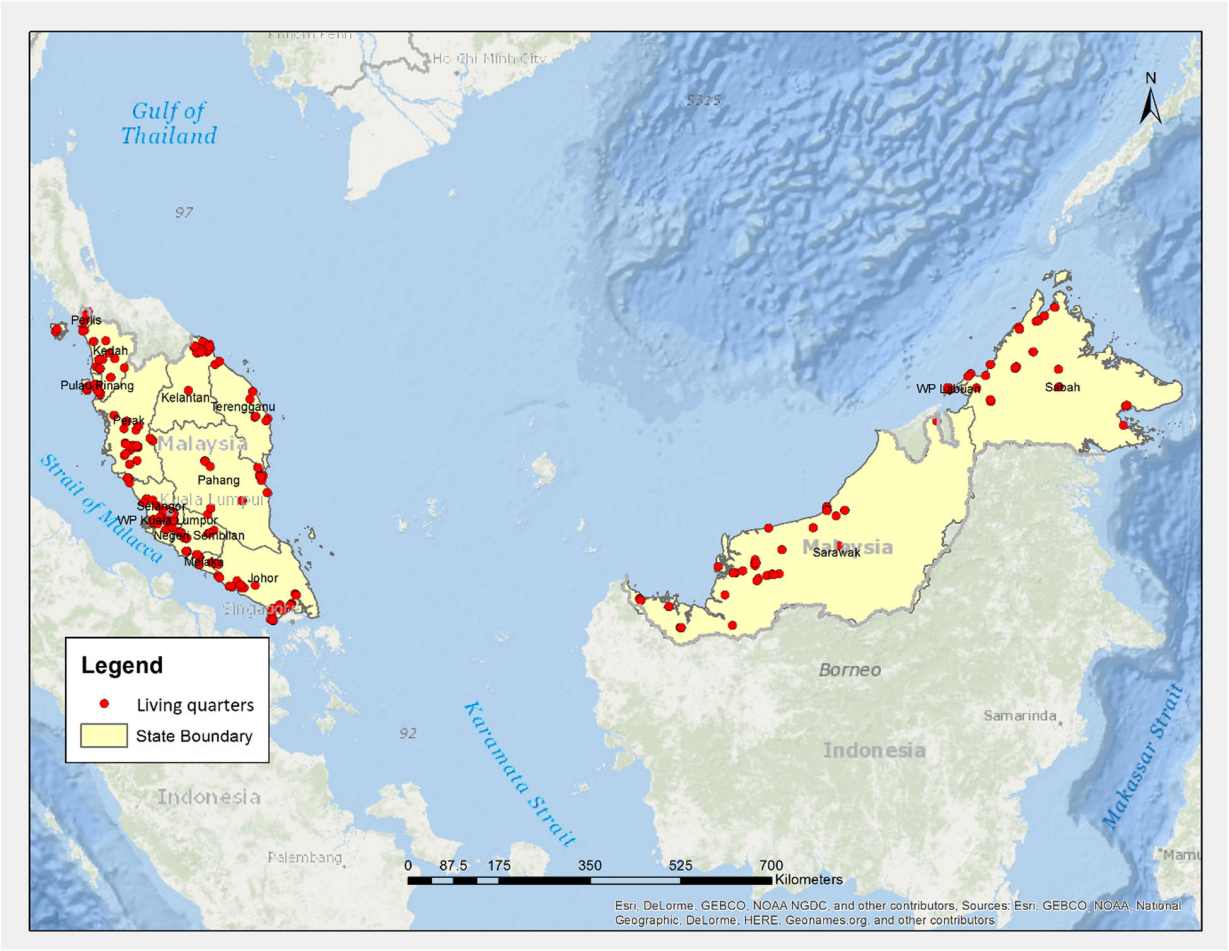
Distribution of selected Living Quarters for the study. (Fig. 1 was created by a Geographic System (GIS) expert from Institute for Public Health, Malaysia, using licensed ArcGIS® software, version 10.3. ArcGIS® and ArcMap™ are the intellectual property of Esri and are used herein under license. Copyright© Esri. All rights reserved. For more information about Esri® software, please visit www.esri.com)

A multi-stage cluster sampling design was used in our study. Respondents were sampled throughout Malaysia (Fig. 1). Geographically, this study covers both urban (strata 1 and 2) and rural areas (strata 3 and 4) in Malaysia. Strata 4 is defined by the Department of Statistics Malaysia as an area with a population of less than 1000 per locality [14]. This study covered 96 EBs throughout Malaysia; 15 LQs were selected from each EB. The first stage of sampling was to select EBs. The second stage was the selection of LQs. LQs from each EB was selected using random probability sampling. The final stage was the selection of eligible residents. A random selection method from a roster of eligible residents was used to select the individuals by gender and age of 18 years and above. When more than one eligible adult was living in the same LQ, only one was selected using a Kish Table. A total of 1047 respondents were identified. The study team interviewed selected respondents to determine eligibility. Ineligible respondents were not replaced.

A complex design study formula was used to calculate the sample size to estimate population prevalence, which includes expected CKD prevalence of 9%, a margin of error of 0.03, and a confidence interval of 95%. Although estimation of CKD prevalence in Malaysia requires an optimal sample size of 700, we targeted 1440 respondents, taking into account a possible high non-response rate due to the biological samples that were needed (venous blood and urine samples).

The inclusion criteria were Malaysians 18 years of age and above who gave written consent for participation in the study. The exclusion criteria were women who were pregnant or menstruating during the data collection. Trained staff conducted face-to-face interviews using tablets with all the respondents to complete the data collection. Measures were taken to minimise the dropout rate and maximise the response rate. Field staff not only worked in the evenings but also on weekends to cater to the working individuals; this even involved visits to the workplace of the respondents.

A sociodemographic questionnaire was used to collect demographics, disease history, and medication use. It was developed by a panel of experts, pre-tested, and validated before the study [15].

Clinical measurements were taken, which included blood pressure (BP), waist circumference, body weight, and height. Blood pressure was measured with a calibrated digital automatic BP monitor (Omron HEM-7221) at chest level with an appropriate-sized cuff. Three BP measurements were taken at 1 to 2-min intervals with the respondent at rest and seated; only the mean of the last two readings was used for analysis. This was done because the first reading of BP in a series is usually the highest [16], and it is recommended that the last two readings in a series be averaged [17] to reflect ones BP truly. Weight was measured to the nearest 0.1 kg with the TANITA model 308 digital weight scale. Height was measured to the nearest 0.1 cm with SECA 307 stadiometer. BMI was categorised based on the WHO guidelines [18]. Waist circumference was measured to the nearest 0.1 cm with a constant tension tape.

Urine albumin-to-creatinine ratio (uACR) was measured using a single urine sample. Urine samples were transported in insulated boxes with ice packs to an accredited central laboratory. Blood for serum creatinine and random blood sugar was taken from respondents at their home by qualified staff from a nearby Ministry of Health (MOH) haemodialysis unit.

**Fig. 2:**
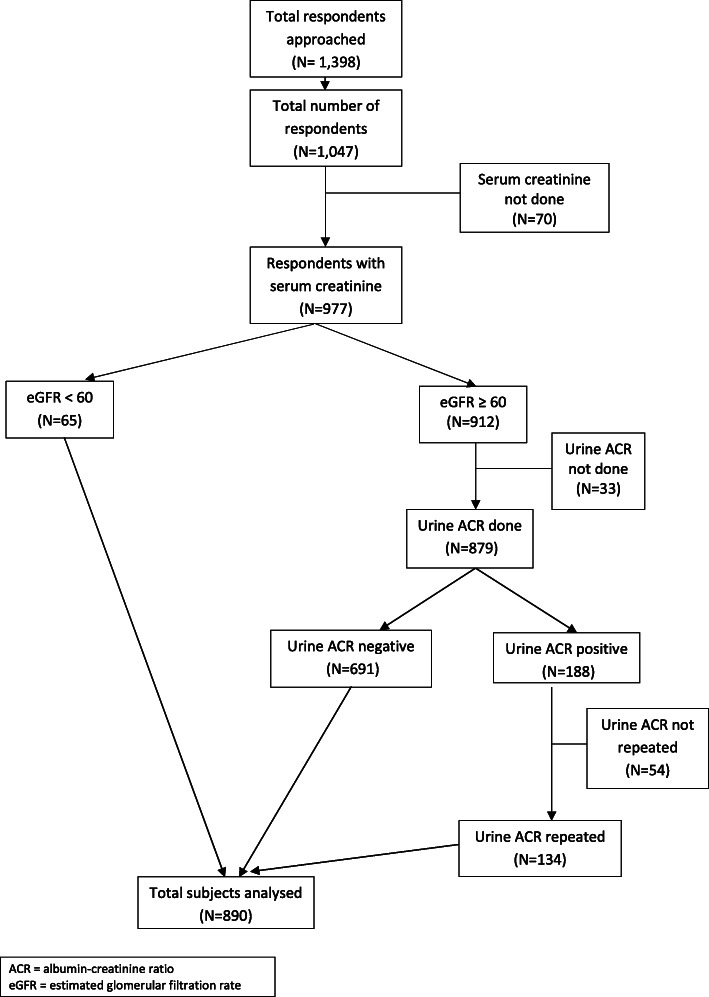
Flow chart of the respondents in the study

Respondents with albuminuria from the initial sampling and eGFR ≥60 ml/m/1.73m^2^ were contacted to repeat uACR within 4 months. Only respondents with albuminuria in repeat sampling were classified as having persistent albuminuria (Fig. 2). Non-persistent albuminuria (positive initial uACR but negative second sample) was classified as normoalbuminuria.

Creatinine content in the urine and serum was measured using the kinetic Jaffe method (kinetic alkaline picrate) with the Abbott Architect ci8200 machine and Abbott/Creatinine reagent. The creatinine assay has calibration traceable to an isotope dilution mass spectroscopy (IDMS). Random blood glucose was measured with the Abbott Architect ci8200 using Abbott/Glucose reagent. Microalbumin in urine was measured using an immunoturbidimetric method.

Completed questionnaires and biochemistry results were sent to the Survey Creation System server centralised in the Institute for Public Health. Datasets were continuously monitored for quality control by the data manager.

The CKD-EPI equation [19, 20] was used to obtain an estimated glomerular filtration rate (eGFR) as follows:

$$ 141\times \mathit{\min}\;{\left( SCr/k, 1\right)}^{\alpha}\times \mathit{\max}\;{\left( SCr/k, 1\right)}^{\hbox{-} 1.209}\times 0.{993}^{Age}\;\left[\times 1.018\; if\kern0.17em female\right], $$

where SCr is serum creatinine (in mg/dl), k is 0.7 for female and 0.9 for male,

α is - 0.329 for female and - 0.411 for male,

min is the minimum of SCr/k or 1, and max is the maximum of SCr/k or 1.

CKD stages were defined as below:

Stage 1 as eGFR > 90 ml/min per 1.73m^2^ and persistent albuminuria;

Stage 2 as eGFR 60–89 ml/min per 1.73m^2^ and persistent albuminuria;

Stage 3 as eGFR 30–59 ml/min per 1.73m^2^;

Stage 4 as eGFR 15–29 ml/min per 1.73m^2^ and,

Stage 5 as eGFR < 15 ml/min per 1.73m^2^.

Kidney damage was assessed using uACR, which provides a sensitive measure for the detection of kidney damage. Based on the *Kidney Disease Improving Global Outcomes (KDIGO) guideline for CKD* [11], albuminuria was defined as below:

Microalbuminuria as urine ACR 30 mg/g – 300 mg/g;

Macroalbuminuria as urine ACR of > 300 mg/g and,

Persistent albuminuria as two episodes of albuminuria at least 1 month apart.

Hypertension, diabetes mellitus, and hypercholesterolemia were defined as self-reported diseases previously diagnosed by medical personnel. Also, blood pressure and random blood sugar levels were tested to detect undiagnosed hypertension and diabetes in those respondents who did not have any previous diagnoses of these two diseases. These respondents were categorised to have hypertension if mean systolic BP was ≥140, diastolic BP was ≥90 mmHg or both [21], and to have diabetes mellitus if the random blood glucose was 11.1 mmol/l [22]. Respondents with abnormal results were referred to the nearest health facility. We did not perform any tests to determine undiagnosed hypercholesterolemia. BMI < 18.5 kg/m^2^ was classified as underweight, 18.5 to 24.9 kg/m^2^ as normal weight, 25 to 29.9 kg/m^2^ as overweight and BMI ≥30 kg/m^2^ as obese. A waist circumference of more than 90 cm in male and more than 80 cm in female were considered as abdominal obesity [23]. The International Physical Activity Questionnaire (IPAQ) Short Version was used to ascertain the level of physical activity [24].

### Statistical methods

Data were analysed using SPSS version 22. Identified outliers were rechecked and verified. The calculated sample weight was according to the Malaysian population aged 18 years and above. The national prevalence of CKD was estimated using complex design analysis. The sampling procedure and the population weight were adjusted for sex and locality (urban/rural) during the analysis [25].

Data were reported as a mean for continuous variables and proportion for categorical variables. Prevalence estimates were performed. Multiple logistic regression was employed to identify factors associated with CKD. The multivariable model was obtained based on the backward stepwise variable selection method. All possible two factors interaction terms were each checked together with the main effects. The fit of the logistic model against the actual outcomes was assessed using the goodness of fit statistics. Adjusted odds ratios (aOR) and 95% confidence intervals (CI) were estimated. A *p*-value of less than 0.05 was considered significant.

## Results

A total of 1398 individuals were approached for this study, and 75% of them (*n* = 1047) consented to participate. Serum creatinine was measured in 977 respondents. Further breakdown of the analysis numbers is shown in Fig. 2. The final analysis set comprised of 890 respondents. There were no significant differences in the characteristics of the overall respondents (*n* = 1047) and the final analysis set.

Table 1 shows the sociodemographic characteristics of our respondents (*n* = 890). Around two-thirds (64.9%) were low-income earners. Nearly half (47.9%) of the respondents had a maximum education level at the secondary school level. Respondents with self-reported hypercholesterolemia were 32.6%, while about half of the respondents (51.0%) had hypertension. More than half (55%) admitted to using pain killers, while 12.6% admitted to using traditional medicines. A total of 31.8% of respondents had ever smoked.

**Table 1:** Characteristics of the CKD study respondents

**Sociodemographic characteristics**	***N*** **= 890**	**%**	**Mean ± SD**
**Age** (years)			48.8 ± 15.6
**Gender**			
Male	366	41.1	
Female	524	58.9	
**Race**			
Malay	590	66.3	
Chinese	87	9.8	
Indian	54	6.1	
Indigenous (East Malaysia) ^a^	147	16.5	
Others	12	1.3	
**Education Attainment**			
No formal education	79	8.9	
Primary	182	20.4	
Secondary	426	47.9	
Tertiary	203	22.8	
**Strata**^**b**^			
Urban	370	41.6	
Rural	520	58.4	
**Income Quartiles**^**c**^			
Low (≤ RM 2613 per month)	578	64.9	
Middle (RM 2614–10,455 per month)	284	31.9	
High (≥ RM 10,456 per month)	28	3.1	
**Systolic BP** (mm Hg)	889		135.6 ± 20.9
**Diastolic BP** (mm Hg)	889		80.0 ± 12.3
**Hypertension**	453	51.0	
**Random Blood Sugar** (mmol/l)	890		6.5 ± 3.7
**Diabetes mellitus**	174	19.6	
**History of Illness**			
Previously diagnosed CKD (*N* = 834)	12	1.4	
Hypercholesterolemia	256	32.6	
(*N* = 785)			
Cardiovascular disease	60	7.1	
(*N* = 841)			
Stroke (*N* = 840)	11.0	1.3	
Family history of CKD (*N* = 871)	83	9.5	
**Painkiller Use in the previous 6 months**	885		
No	398	45.0	
Yes	487	55.0	
At least once a day	27	3.0	
At least once a week	21	2.4	
At least once a month	1	0.1	
Less than once a month	372	42.0	
Not specified	66	7.5	
**Traditional Medicine**	883		
No	772	87.4	
Yes	111	12.6	
At least once a day	55	6.2	
At least once a week	19	2.2	
At least once a month	19	2.2	
Less than once a month	16	1.8	
Not specified	2	0.2	
**Cigarette Smoking**	890		
Ever smoker	283	31.8	
Current smoker	174	61.5	
Non-current smoker	109	38.5	
Never smoker	607	68.2	
**Physical Activity**	890		
Active	533	59.9	
Inactive	357	40.1	
**BMI**	890		
Underweight (< 18.5 kg/m^2^)	39	4.4	
Normal (18.5–24.9 kg/m^2^)	313	35.2	
Overweight (25.0–29.9 kg/m^2^)	319	35.8	
Obesity (> 30.0 kg/m^2^)	219	24.6	
**Waist circumference (cm)**	890		
Male	366		90.8 ± 12.2
Female	524		89.1 ± 13.2
**Abdominal obesity**^**d**^	890		
Yes	588	66.1	
No	302	33.9	

^a^The states of Sabah and Sarawak are in northern Borneo

^b^Urban = population ≥ 10,000 per enumeration block (EB), rural = population 1000–10,000 per EB

^c^in 2018, 1 USD = 4.0 RM

^d^Waist circumference > 90 cm in males and > 80 cm for females

Out of the 890 respondents 58.0% had normal renal function, 35.2% had mildly decreased renal function (eGFR 60–89 ml/min/1.73m^2^) and 6.8% had moderate to severe renal failure (eGFR < 60 ml/min/1.73m^2^) (Table 2).

**Table 2:** Prevalence of eGFR by categories (*N* = 890)

**eGFR** **(ml/min/1.73 m**^**2**^**)**	**Count** **(n)**	**Estimated population**	**Prevalence** **(%)**	**95% CI**
Normal (≥90)	507	9,765,081	58.0	52.19, 63.57
Mildly decreased (60–89)	318	5,927,978	35.2	30.30, 40.44
Moderately to severely decreased (< 60)	65	1,146,526	6.8	4.71, 9.76

*eGFR* estimated glomerular filtration rate in ml/min/1.73 m^2^ using the CKD-EPI equation

Albuminuria was detected in 16.9% of the sample (Table 3). Among the 174 (19.6%) respondents who had DM in the study, 37.3% had albuminuria. Albuminuria was three times higher in the diabetic group compared to the non-diabetic group (11.4%).

**Table 3:** Prevalence of albuminuria by diabetes status

Albuminuria	All (*N* = 890)		Diabetes (*N* = 174)		No Diabetes (*N* = 716)	
	Count	% (95% CI)	Count	% (95% CI)	Count	% (95% CI)
Normoalbuminuria	715	83.1 (79.58, 86.09)	99	62.6 (49.7, 74.0)	616	88.6 (85.63, 91.02)
Microalbuminuria^a^	146	13.7 (11.02, 16.90)	57	28.4 (19.2, 39.9)	89	9.7 (7.27,12.89)
Macroalbuminuria^b^	29	3.2 (2.08, 4.96)	18	8.9 (4.5, 17.0)	11	1.7 (0.86, 3.25)

^a^Microalbuminuria is 30–300 mg/g albumin

^b^Macroalbuminuria is > 300 mg/g albumin

Repeat measurement of albuminuria was performed in 134 respondents (Table 4) with eGFR ≥60 ml/min/1.73m^2^. Albuminuria persisted in 66.5% among those with microalbuminuria and 89.9% among those with macroalbuminuria.

**Table 4:** Prevalence of persistent albuminuria

**Albuminuria**	**TOTAL**	**Normoalbuminuria in the second specimen**		**Microalbuminuria in the second specimen**		**Macroalbuminuria in the second specimen**	
	***n*** **= 134**	**n**	**% (95% CI)**	**n**	**% (95% CI)**	**n**	**% (95% CI)**
**Microalbuminuria in first specimen**	115	39	33.6 (21.23, 48.66)	67	55.9 (40.65, 70.05)	9	10.6 (4.61, 22.43)
**Macroalbuminuria in first specimen**	19	2	10.1 (1.97, 38.56)	9	37.5 (15.07, 66.92)	8	52.4 (24.62, 78.81)

The adjusted prevalence of CKD is shown in Table 5. The prevalence of CKD stage 1 was 3.85%, stage 2 was 4.82%, stage 3 was 6.48%, stage 4 was 0.25%, and stage 5 was 0.08%. The overall CKD prevalence was 15.48%. Of the 158 respondents with CKD, only 8 (5%) were aware of their disease. By locality, the prevalence of CKD in the urban area was 14.2% (95%CI: 10.37, 19.05), while in the rural area, it was 20.0% (95%CI: 15.84, 24.82).

**Table 5:** Prevalence of CKD by stages (*N* = 890)

**CKD Stages**	**n**	**Estimated population**	**Prevalence (%)**	**95% CI**
**Total CKD**	158	2,607,448	15.48	12.30, 19.31
**Stage 1**	42	649,069	3.85	2.51, 5.87
**Stage 2**	51	811,853	4.82	3.14, 7.32
**Stage 3**	59	1,091,582	6.48	4.41, 9.43
**Stage 4–5**	6	54, 944	0.33	0.14, 0.78

Using multivariate analysis*,* we found that hypertension, diabetes, increasing body mass index (BMI), and increasing age were significantly associated with increased risk for CKD (Table 6). Individuals who had diabetes were 3.3 times more likely to have CKD while the likelihood increased by 3.7 times in those with hypertension.

**Table 6:** Factors associated with chronic kidney disease by univariate and multivariate analysis (*N* = 890)

**Variable**	**N**	**% with CKD**	***p*-value**	**Unadjusted OR** **(95% CI)**	***p*-value**	**Adjusted OR**^**a**^ **(95% CI)**
**Age (years)**			< 0.001	1.08 (1.06,1.09)	**< 0.001**	**1.06 (1.04,1.08)**
**Gender**						
Male	59	16.1		1		1
Female	99	18.9	0.287	1.21 (0.85,1.73)	0.198	1.50 (0.81, 2.79)
**Race**						
Malay	110	18.6		1		1
Chinese	11	12.6	0.176	0.63 (0.33, 1.23)	0.196	0.60 (0.27, 1.31)
Indian	7	13.0	0.303	0.65 (0.29,1.48)	0.204	0.53 (0.20, 1.42)
Others	30	18.9	0.949	1.02 (0.65, 1.59)	0.153	1.53 (0.86, 2.73)
**Strata**						
Urban	56	15.1		1		1
Rural	102	19.6	0.085	1.37 (0.96,1.96)	0.141	1.42 (0.89,2.27)
**Household Income**						
Low (<RM 2614 per month)	112	19.4		1		1
Middle (RM 2614–10,455 per month)	42	14.8	0.506	1.44 (0.49,4.24)	0.133	1.48 (0.89, 2.45)
High (≥RM 10456 per month)	4	14.3	0.943	1.04 (0.34,3.15)	0.307	1.93 (0.55, 6.87)
**Ever Cigarette Smoking**						
Yes	47	16.6	0.542	0.89 (0.61,1.30)	0.251	1.46 (0.77, 2.78)
No	111	18.3		1		1
**Physical Activity**						
Active	83	15.6		1		1
Inactive	75	21.0	0.038	1.442 (1.02,2.04)	0.368	1.23 (0.79, 1.91)
**Diabetes**						
Yes	80	46.0	< 0.001	6.96 (4.76,10.18)	**< 0.001**	**3.32 (2.20,5.03)**
No	78	10.9		1		**1**
**Hypertension**						
Yes	141	31.1	< 0.001	11.14 (6.60,18.81)	**< 0.001**	**3.72 (2.08,6.66)**
No	17	3.9		1		**1**
**Hypercholesterolemia**						
Yes	79	30.9	< 0.001	3.08 (2.13,4.45)	0.513	1.17 (0.73, 1.87)
No	67	12.7		1		1
**Heart Disease**						
Yes	18	30.0	0.017	2.03 (1.14,3.64)	0.152	0.58 (0.27, 1.22)
No	136	17.4		1		1
**Family History of Kidney Disease**						
Yes	17	20.5	0.521	1.20 (0.68,2.11)	0.099	1.89 (0.89, 4.01)
No	139	17.6		1		1
**Painkiller Use**						
Yes	80	16.4	0.221	0.81 (0.57,1.14)	0.723	1.08 (0.70, 1.68)
No	78	19.6		1		1
**Traditional Medicine Use**						
Yes	17	15.3	0.468	0.82 (0.47,1.41)	0.281	0.69 (0.35,1.36)
No	140	18.1		1		1
**BMI (kg/m**^**2)**^			< 0.001	1.07 (1.03,1.10)	**0.006**	**1.06 (1.02,1.10)**
**Abdominal obesity**						
Yes	123	20.9	0.001	2.02 (1.35,3.02)	0.260	0.68 (0.35, 1.33)
No	35	11.6	1			1

Definition of chronic kidney disease status: eGFR< 60 ml/min/1.73 m^2^ and/or albuminuria

*OR* Odds Ratio, *CI* Confidence Interval, *eGFR* estimated glomerular filtration rate, *BMI* Body Mass Index

^a^Final model was adjusted by age, diabetes, hypertension, and BMI

## Discussion

The CKD prevalence in Malaysia in 2018 was 15.48%, which is similar to other countries in Asia. The prevalence of CKD in the region varies, from 17.5% in Thailand [26], 17.2% in India [27], 15.6% in Singapore [28], 10.8% in China [29]. However, there were differences in the CKD definition, study design, and methodology.

This study has demonstrated a rising prevalence of CKD in Malaysia over the last 7 years since the previous study, the prevalence of 9.07 to 15.48%. The main reasons accounting for this rising trend are the increasing prevalence of non-communicable diseases and also changes in population demographics. National Health Morbidity Surveys have shown an alarming increase in the prevalence of diabetes in Malaysia over the past decade; from 11.6% in 2006 to 15.2% in 2011 and 17.5% in 2015; with the prevalence of hypertension persistently high at above 30.0% in the same surveys [8, 30, 31]. The prevalence of overweight and obesity among adults had also increased during the same period. Prevalence of obesity was 14.0% in 2006, 15.1% in 2011 and 17.7% in 2015 [8, 30, 31]. Population ageing in Malaysia could also have contributed to the observed increase in CKD prevalence as the median age of the overall Malaysian population was 26.3 years in 2010 [32] and 28.6 in 2018 [33].

Besides, there were several significant differences compared to the previous study in 2011. The prevalence of CKD was weighted to population size, age, sex, and urban/rural residents in this study using complex sampling design analysis; this was not performed in the previous study. Also, the previous study covered only West Malaysia, while this study included the whole of Malaysia, including Sabah and Sarawak (East Malaysia). In Malaysia, the distribution of urban to rural population was 75.5 and 24.5% in 2017 [34]. In the earlier study, the rural population was under-represented due to logistic issues (9.7% in 2011 versus 54.8% in this study). The proportion of low-income groups was lower in 2011 (35.7%) compared to this study (64.9%). Also, the mean age (48.8 years) of respondents in this study was 5.9 years older compared to the previous study (42.9 years).

Our study shows that awareness of CKD diagnosis was still low at 5%, which is similar to the previous study in Malaysia (4%). Awareness of CKD was found to be 12.5% in China [29], 7.9% in India [27], 5.3% in Canada [35] and 1.9% in Thailand [26]. An extremely low awareness level of CKD is worrying as this could imply late detection and thus missed opportunities to prevent more serious complications in the future. A concerted effort to educate the public, as well as health care providers, is necessary to increase awareness of CKD, especially in primary healthcare settings which can target high-risk groups.

This study also found that respondents with diabetes had a three times higher incidence of albuminuria compared to non-diabetics (37.3% versus 11.4%). The incidence of new dialysis patients with diabetes mellitus in Malaysia has risen from 44% in 2000 to 65% in 2016 [9]. In light of this finding, more effort is required to ensure health care providers adhere strictly to clinical practice guidelines (CPG), which recommend annual screening for albuminuria, achieving optimal glycaemic control and treatment with renin-angiotensin blockers for those with albuminuria [20]. This can assist in the prevention, early detection, and treatment of CKD to delay progression to end-stage renal failure.

Studies on CKD prevalence in other countries found that CKD was closely associated with diabetes mellitus, increasing age, obesity, hypertension [36–38], use of nonsteroidal anti-inflammatory drugs and traditional medicines [39]. In this study, diabetes mellitus, hypertension, BMI, and increasing age were identified as significant associated factors. These high-risk groups must be prioritised in our health care setting for CKD screening activities. This will enable early detection and institution of appropriate measures to increase the possibility of slowing or even halting progression to the next CKD stage and also to reduce its possible cardiovascular complications [5, 6, 40].

The strengths of this study include the use of an accredited central laboratory, the use of standardised equipment, trained workforce and questionnaires that were validated by the previous NHMS studies [15]. The results were adjusted by complex analysis to represent the general population [25].

On the other hand, this study also has several limitations. First, an actual cause and effect relationship could not be established in this study due to its cross-sectional design. Secondly, we did not include very rural areas in this study, especially in East Malaysia, due to logistic reasons. Thirdly, kidney damage was only assessed using a single parameter, which was albuminuria. If other signs of kidney damage (e.g., persistent haematuria/ ultrasound structural changes) were included in this study, the prevalence of CKD might have been higher. Although we managed to repeat uACR testing to confirm persistent albuminuria, the same could not be done for serum creatinine after 3 months due to logistic limitations. Thus, some subjects with acute kidney injury could have been included in this study.

## Conclusions

Our study has shown that CKD has become one of the leading public health issues in Malaysia with increased prevalence and low awareness. Our results support the need to emphasise on improving the education and early detection of CKD in the community. The findings of this study have helped to convince policymakers of the need to stem the rising tide of CKD in the country. In April 2018, the National Strategic Action Plan [41] adopted CKD prevention and management initiatives (ACT-KID 2018–2025). This action plan includes stakeholders in primary care, public health, nephrology, professional societies, and non-governmental organisations. It aims to improve all levels of CKD care from prevention and early detection to the other end of the spectrum, i.e., renal replacement options. Future studies to re-assess the trends in CKD prevalence will be necessary to gauge the impact of these initiatives.

## Data Availability

The datasets generated or analysed during the current study are not publicly available as per the research protocol of the Institute for Public Health Malaysia as it contains information that could compromise research participants’ privacy/consent but are available from the corresponding author on reasonable request.

## Abbreviations

BMI: Body mass index
BP: Blood pressure
CKD: Chronic kidney disease
DM: Diabetes mellitus
EB: Enumeration blocks
LQ: Living quarters
NHMS: National Health and Morbidity Survey
uACR: Urine albumin-to-creatinine ratio
